# Probiotics mitigating subclinical necrotic enteritis (SNE) as potential alternatives to antibiotics in poultry

**DOI:** 10.1186/s13568-020-00989-6

**Published:** 2020-03-14

**Authors:** Abdul Khalique, Dong Zeng, Muhammad Shoaib, Hesong Wang, Xiaodan Qing, Danish Sharafat Rajput, Kangcheng Pan, Xueqin Ni

**Affiliations:** 1 Animal Microecology Institute, College of Veterinary, Sichuan Agricultural University, Chengdu, 611130 China; 2 Key Laboratory of Animal Disease and Human Health of Sichuan Province, Chengdu, Sichuan China; 3 Department of Microbiology, Faculty of Biology, Baku State University, Baku, Azerbaijan

**Keywords:** *Clostridium perfringens*, Probiotics, Subclinical necrotic enteritis, *Bacillus*, *Lactic acid bacteria*, Antimicrobial growth promoters

## Abstract

Subclinical necrotic enteritis (SNE) caused by *Clostridium perfringens* (*CP*), is an important disease in chickens, which causes huge economic losses by damaging the intestinal mucosa, decreasing digestion and absorption of nutrients. Use of antibiotics at a sub-therapeutic level as antimicrobial growth promoters in poultry feed prevents the birds from SNE and improves growth. Due to the ban on the use of antibiotics in 2006 as antimicrobial growth promoters have led to the reemergence of the disease. Worldwide numerous studies have been carried out to investigate the alternatives to antibiotics for the prevention of SNE. Possible alternatives to control SNE include probiotics, prebiotics, bacteriophages, essential oils, organic acids, secondary metabolites and other microbial products. Currently, probiotics are most extensively used in poultry production as an alternative to antibiotics. This review summarizes recent insights and experimental evidence on the use of different microorganisms like *Bacillus, Lactic acid bacteria, Bifidobacteria, Enterococcus, yeast*, etc. as valuable probiotics for prevention of SNE and potential molecular mechanisms responsible for ameliorating effects of probiotics against SNE.

## Introduction

In broilers, subclinical necrotic enteritis (SNE) is caused by *Clostridium perfringens* (*CP*), which is also responsible for causing numerous diseases like enterotoxaemia and gangrenous dermatitis in animals. It is Gram-positive anaerobic bacteria, which produces spores and highly virulent toxins (Kay et al. 2019; Tian et al. 2016). This is commonly found in many environmental sources like soil, sewage, litter, feces of chicken as well as in the intestine of humans and animals (Songer 1996). This bacterium has zoonotic importance as it causes foodborne diseases in humans, but it also poses an important threat for animals (Grass et al. 2013; Wakabayashi et al. 2019). Outbreaks of necrotic enteritis (NE) usually peaks between 2 and 6 week of age (Hunter et al. 2019; Yang et al. 2016). Due to the subclinical nature of the disease, there is chronic damage to the intestinal mucosa of the chickens (Wu et al. 2019; Zhang et al. 2019). This leads to impaired absorption of nutrients, reduced weight gain and decreased overall performance (Antonissen et al. 2014; Skinner et al. 2010). Due to these factors, subclinical necrotic enteritis causes great economic loses to the poultry industry, which is estimated to be 2 billion dollars per year (Khalique et al. 2019; Timbermont et al. 2011).

*CP* is usually found in the intestines of healthy chicken less than 10^5^ cfu/g intestinal content (Calik et al. 2019; Hernandez-Patlan et al. 2019). If *CP* counts are more, i.e. 10^6^ cfu/g of digesta, in the small intestine of chickens, then birds become prone to NE (Kiu and Hall 2018; Yang et al. 2016). There are many predisposing factors, such as viscosity of digesta, the presence of non-digestible polysaccharides, the gastrointestinal tract transit time and the intestinal pH, which are responsible for the ability of bacteria to cause the disease (Moore 2016; Yang et al. 2019). One of the important factors is the coccidiosis caused by *Eimeria* species, which creates a suitable environment for the proliferation of *CP* (Si et al. 2007; Van Waeyenberghe et al. 2016). Due to coccidiosis, perforations are made in the intestinal epithelial lining, and plasma proteins accumulate in these holes. These proteins are utilized as a substrate by *CP,* and this leads to the development of disease (Van Immerseel et al. 2009). Any factor which induces stress in chickens disrupts the balance of microbial flora of intestine and suppresses immune system contributes to the risk of SNE (Tsiouris 2016).

Toxins are main virulence factors of *CP,* and there are 17 different types of toxins produced by *CP*, which include α, β, β2, ε, ι and the enterotoxin CPE (Gkiourtzidis et al. 2001; Kumar et al. 2019). Based on toxin production, isolates of *CP* are divided into five main groups (Uzal et al. 2014). Type A *CP* is mainly responsible for SNE in poultry by producing α toxin and the pore-forming toxin NetB. Mainly α toxin causes NE, but many reports have shown that NetB toxin alone can also cause the disease (Keyburn et al. 2010). The α toxin has 370 amino acid residues and is Zinc Metallo Phospholipase. Phosphatidylcholine and sphingomyelin in the eukaryotic cell membrane are the target of α toxin and lead to lysis of cells. Low concentrations of α toxin cause limited membrane damage and accumulation of diglycerol. This leads to activation of the arachidonic acid pathway and hence release of inflammatory mediators (Riaz et al. 2017). NetB toxin induces the formation of pores in eukaryotic cell membranes and thus causes cytotoxicity (M’Sadeq et al. 2015). The sequencing of genes encoding NetB toxin has shown that this coding sequence is highly conserved across all the strains of *CP*, which causes NE. Genomic analysis has shown that genes, in addition to NetB toxin, are also associated with NE causing by *CP* strains (Lacey et al. 2016). In addition to toxins, *CP* also produces bacteriocins, which play an important role in the virulence. These bacteriocins inhibit the growth of non-pathogenic microbes for the competition of nutrients that create an imbalance in intestinal microflora. Perfrin is one of the important bacteriocins produced by *CP* (Timbermont et al. 2014). The ability of pathogenic strains of *CP* to inhibit the proliferation of normal intestinal strains is one of the major reasons for the development of NE. Bacteriocins help the pathogenic strains of *CP* to replace the normal intestinal flora of chickens (Riaz et al. 2017). Hydrolytic and proteolytic enzymes produced by *CP* destroy basal lamina and lateral domains of intestinal cells. Due to increased activity of these enzymes, especially collagenolytic activity, there are morphological changes and mucosal damage, which play a very important role in development of NE (Olkowski et al. 2008).

Antibiotics had been most commonly used as growth promoters and for prophylaxis of SNE (Prescott et al. 2016). Residues of these antibiotics in the chicken meat have harmful effects on the health of human beings, i.e., cause of antibiotic resistance. There is a ban on the use of antibiotics as feed additives in Europe in 2006 under feed additives regulation 1831/2003/EC (Kemper 2008). To prevent the economic losses caused by SNE, there is a dire need for some alternatives to antibiotics. Probiotics, prebiotics, plants, molecules of plant origin, organic acids, enzymes, lysozyme or molecules of microbial origin, such as yeast extract and antimicrobial peptides are different substances, which can be used as alternatives to antibiotics (Caly et al. 2015). Whereas, probiotics have been used widely in the poultry industry as a potential alternative to antibiotics. This review summarizes the usefulness and effectiveness of different probiotic strains as alternatives to antibiotics for the prevention of SNE.

### Prophylactic use of probiotics in poultry feed

**Fig. 1:**
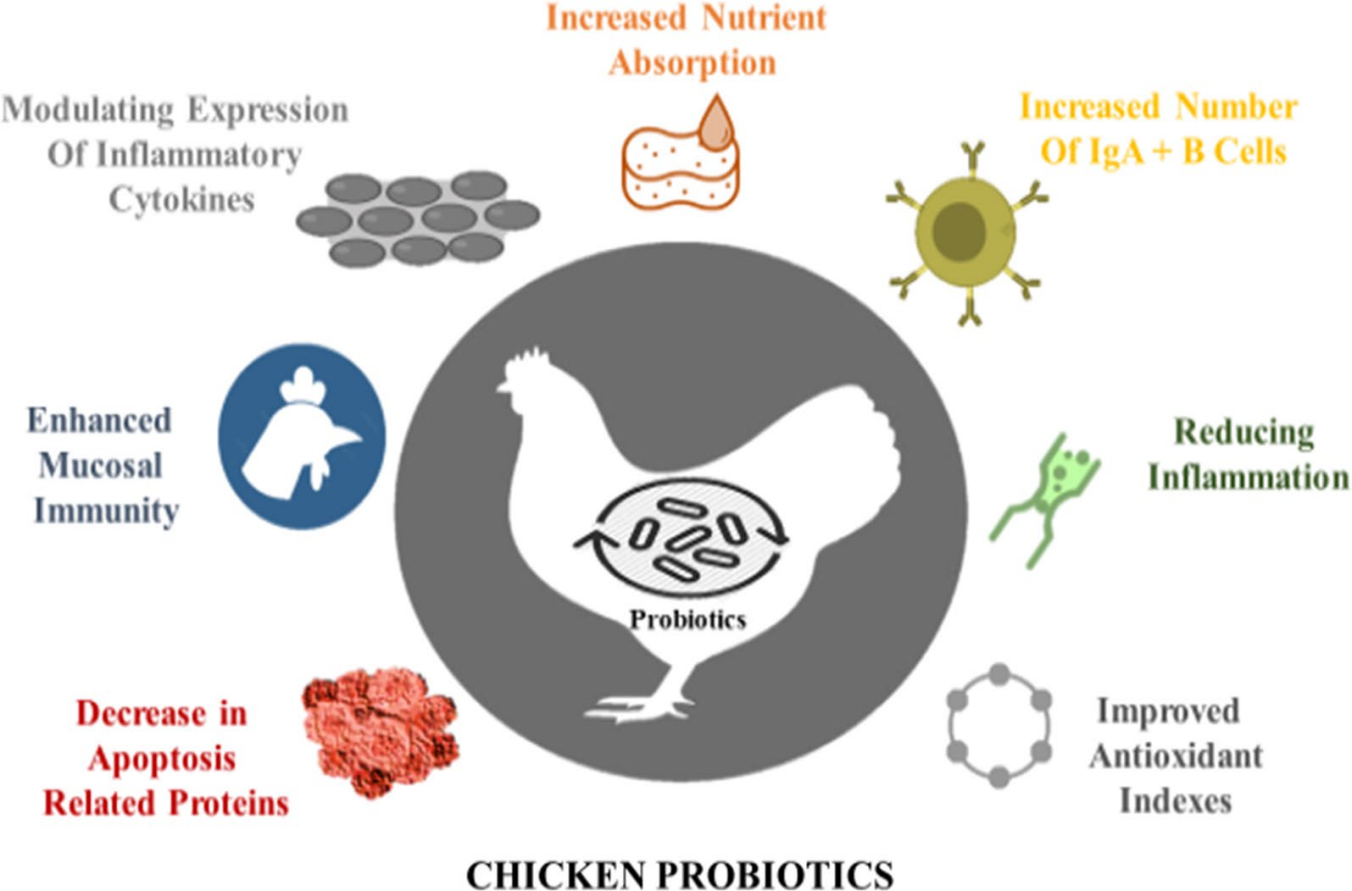
Effects of probiotics on intestinal health of chickens

Probiotics are defined as live microbial supplements, which are used in the feed to improve the health of animals by balancing the intestinal microbes. Supplementation of single bacterial strain or mixture of different bacterial strains or yeast prevents the growth of pathogens in intestine. It not only decreases the incidence of intestinal diseases but also improves the overall performance of birds (Chaucheyras-Durand and Durand 2009). Probiotics reduce the risk of SNE by enhancing host immunity, improving the intestinal microflora balance and stimulating metabolism. These probiotics also produce antimicrobial substances, which inhibit the growth of pathogenic bacteria (Pan and Yu 2014). Probiotics also compete with pathogenic bacteria in the intestine of chickens for growth and nutrients, which is known as competitive exclusion (Caly et al. 2015). Possible mode of actions of probiotics includes competitive exclusion, increased digestive enzyme activity, production of substances that can inhibit the growth of pathogens or neutralize enterotoxins, modulation of the host’s immune development, and alteration of intestinal microbial activity (Sokale et al. 2019). Numerous mechanisms of action of bacterial and yeast probiotic have been proposed. These include the production of organic acids, bacteriocins and H_2_S, competition for nutrients, anti-inflammatory properties and inhibition of pathogen adhesion to the epithelium. Furthermore, these probiotics are also responsible for modulation of the immune system, interference on bacterial-induced signaling pathways, and actions on bacterial virulence factors (Vieira et al. 2013). Effects of probiotic on intestinal health of chicken are shown in (Fig. 1).

### Prevention of SNE by the composition of broiler intestinal flora

The composition of poultry intestinal microflora plays an important role in the development and prevention of SNE induced by *CP*. For example, butyrate-producing bacteria belonging to the *Lachnospiraceae* family inhibit the inflammation in the intestine of poultry and preserve the intestinal activity. Lactic acid bacteria promote the colonization of butyrate-producing bacteria. Similarly, *Candidatus savagella* stimulates Th17 cells and induces the formation of immunoglobulin A. Hence these *Lactic acid bacteria* and *Candidatus* spp. can be used as potential probiotics (Antonissen et al. 2016). Numerous studies have shown anti-*CP* activity of various microorganisms, i.e., genera *Bacillus*, *Lactobacilli*, *Enterococci*, *Bifidobacteria*, and yeasts (Table 1).

**Table 1:** Different microorganisms used as probiotics for the prevention of subclinical necrotic enteritis (SNE)

Bacteria	Species	References
Bacillus	*Bacillus cereus 8A, Bacillus licheniformis, Bacillus pumilus, Bacillus subtilis, Bacillus coagulans*	Bizani and Brandelli (2002), Barbosa et al. (2005), Klose et al. (2010), Jayaraman et al. (2013), Zhou et al. (2016), Khan et al. (2016)
Lactic acid bacteria	*Lactobacillus acidophilus, Lactobacillus amylovorus, Lactobacillus animalis, Lactobacillus fermentum, Lactobacillus johnsonii FI9785, Lactobacillus johnsonii BS15, Lactobacillus mucosae, Lactobacillus plantarum, Lactobacillus reuteri, Lactobacillus salivarius*	Cao et al. (2012), Caly et al. (2015), Allaart et al. (2011), Qing et al. (2017), Wang et al. (2018), Lin et al. (2008), Guo et al. (2017)
Enterococci	*Enterococcus faecium, Enterococcus faecalis, Enterococcus durans*	Klose et al. (2010), Fukata et al. (1991), Caly et al. (2015)
Bifidobacteria	*Bifidobacteria animalis sub species lactis, Bifidobacteria thermoacidophilum*	Schoster et al. (2013), Klose et al. (2010)
Yeast	*Saccharomyces cerevisiae, Candidatus savagella*	Layton et al. (2013), Eeckhaut et al. (2016)

### *Bacillus* species as probiotics for prevention against SNE

Several species of genus *Bacillus* have shown anti-*CP* activity. These species include *Bacillus cereus 8A* (Bizani and Brandelli 2002), *Bacillus licheniformis* and *Bacillus pumilus* (Barbosa et al. 2005) and *Bacillus subtilis* (Jayaraman et al. 2013; Klose et al. 2010). *Bacillus* spp. as probiotics promote the growth of broilers affected with NE by improving weight gain and feed efficiency (Lee et al. 2014). *Bacillus* spp. not only regulate the fatty acid synthesis and oxidation-related genes in the livers of *CP* affected chickens but also enhance antioxidant activity (Zhou et al. 2016). *Bacillus* spp. produce bacteriocins which inhibit the growth of *CP*. *Bacillus subtilis strain SP6* has shown in vitro anti-*CP* activity. This activity was due to production of heat-stable protein substances. When *Bacillus subtilis strain SP6* was used for the treatment of SNE infected chicken, it markedly reduced the mortality by improving the intestinal health of chickens (Teo and Tan 2005). *Bacillus* spores, used in the poultry feed, could increase the performance of chickens and reduce mortality in chickens affected with NE (Knap et al. 2010). Supplementation of *Bacillus* spp. in the *CP* affected chicken’s diet increased serum nitric oxide levels and decreased Coccidia by specific antibodies (Lee et al. 2014).

*Bacillus subtilis,* when used as probiotics, improved the microbial balance in the intestine of chickens by immune stimulation and competitive exclusion (Khan et al. 2016). *Bacillus subtilis* having a broad activity against *Clostridium* spp. inhibits the pathogen and improves overall performance in broilers (Abudabos et al. 2017). *Bacillus subtilis* can support the reduction of *C. perfringens* in the ileum and caecum positively by altering the intestinal microbial population, and supporting the improvement of growth performance (Bortoluzzi et al. 2019; Whelan et al. 2018). *Bacillus subtilis* has the ability to improve NE-associated pathology and performance of broiler chickens (Jayaraman et al. 2013; Musa et al. 2019). The diet of broiler chickens, challenged with *CP*, was supplemented with *Bacillus subtilis* as probiotic, which showed improvement of serum biochemical profiles of chickens. Moreover, there was not only a decrease in the triglyceride and total cholesterol content in serum but also a significant increase in the number of lymphocytes. This study demonstrated the potential of *Bacillus subtilis* as probiotic for prevention of NE (Al-Baadani et al. 2018). Dietary supplementation of *Bacillus subtilis* DSM 32315 ameliorated the pathological conditions and performance detriments associated with NE. Birds supplemented with *Bacillus subtilis* DSM 32315 exhibited better body weight, lower mortality and intestinal NE lesion score as compared to the non-supplemented birds. Furthermore, histomorphometry of intestine showed shallower crypt depth and higher villus height to crypt depth ratio (Bortoluzzi et al. 2019).

The effects of *Bacillus licheniformis* as a dietary supplement were evaluated on growth performance, and the lipid metabolism genes in the chickens with *CP* induced NE. It was found that *Bacillus licheniformis* as probiotic in chickens with NE can enhance growth performance as well as alter the expression of the gene in fatty acid and lipid metabolism (Zhou et al. 2016). Chickens challenged with* CP* have a disruption in the microflora of caecum. *Bacillus licheniformis* supplementation in the diet of chickens improved the microbial balance by alleviating the disruption of caecal microbiota and improved the growth performance. This shows that *Bacillus licheniformis* can be used as probiotics for prevention of SNE (Lin et al. 2017). The diet of chickens infected with NE was supplemented with *Bacillus licheniformis*, which led to normalization of ileum microflora of chickens (Xu et al. 2018).

*Bacillus coagulans* promotes the growth performance of chickens and increases the feed digestibility by secreting enzymes such as protease, α-amylase, xylanase and lipidase. It also produces amino acids and vitamins (Hung et al. 2012). *Bacillus coagulans*, when used as probiotics, enhances the immunity of chickens and decreases gut inflammation (Fitzpatrick 2013). The influence of feeding *Bacillus coagulans* on the growth rate and intestinal health status of chickens with *CP* induced necrotic enteritis was investigated. *CP* challenged birds were fed diets supplemented with *Bacillus coagulans* exhibited lower gut lesion scores and decreased *CP* numbers in the caecum and liver. It also improved the growth performance and inhibited colonization and invasion of * CP* (Al-Baadani et al. 2018).

### Lactic acid bacteria as probiotics for prevention against SNE

Lactic acid bacteria can be used as probiotics as an alternative to antibiotics due to their antimicrobial properties, as well as beneficial effects on the host. Probiotic potential of Lactic acid bacteria is attributed to the production of bacteriocins, lactate and hydrogen peroxide, and boosting the immunity of the host. These microbes increased the expression of cytokines in the host, thus helping to boost stronger immune response (Cao et al. 2012). As reported by Caly et al. (2015), that different species of the Lactic acid bacteria can be used as potential probiotics. These species include *Lactobacillus acidophilus, Lactobacillus amylovorus, Lactobacillus animalis, Lactobacillus fermentum, Lactobacillus johnsonii FI9785, Lactobacillus mucosae, Lactobacillus plantarum, Lactobacillus reuteri* and *Lactobacillus salivarius. Lactobacillus fermentum* inhibited the β2 toxin production by CP, and this phenomenon occurred at the transcriptome level (Allaart et al. 2011).

*Lactobacillus johnsonii*, when fed to 20-day old chickens, was able to persist in the intestine and after 15 days it inhibited the colonization of *CP* (La Ragione et al. 2004). *Lactobacillus johnsonii BS15* was used as probiotic in broilers for prevention of SNE. This strain not only ameliorated the SNE induced average daily weight loss but also improved the liver abnormalities. *Lactobacillus johnsonii BS15* ameliorated the lipid metabolism and improved the intestinal microflora of chickens. Thus, *Lactobacillus johnsonii BS15* can be used as potential probiotic for prevention of SNE (Qing et al. 2017). *Lactobacillus johnsonii BS15* improved the blood parameters related to immunity in chickens infected with SNE. The *Lactobacillus johnsonii BS15* not only increased antioxidant capacities but also enhanced the serum levels of antibodies, interleukin-2 and interferon-gamma. This showed that *Lactobacillus johnsonii BS15* could improve the immunity of birds infected with SNE (Wang et al. 2018). Hepatic transcriptome analysis showed that *Lactobacillus johnsonii BS15* as a probiotic improves the lipid metabolism in broilers infected with SNE (Allaart et al. 2011). *Lactobacillus johnsonii* BS15 also decreased liver inflammation by regulating different inflammatory pathways and genes in the broiler chicken (Table 2) (Khalique et al. 2019).

**Table 2:** Regulatory pathways and gene modifications by *Lactobacillus johnsonii BS15* decreased liver inflammation

Pathway	Genes
Cytokine-cytokine receptor interaction	CCR2; CX3CR1; IFNGR1; IL18; IL1B; IL2RA; IL2RG; TNFRSF1B; TNFRSF21; TNFSF11; XCL1; XCR1; CNTFR; GHR; IL12RB1; LIFR
Intestinal immune network for IgA production	CD80; HLA-DRA; ITGA4
Toll-like receptor signaling pathway	CD80; IL1B; PIK3R5; TLR2A; TLR4; FOS
Phagosome	CTSS; HLA-DRA; MARCO; MRC2; TLR2A; TLR4, NCF2; TUBB6
Influenza A	HLA-DRA; IFNGR1; IL18; IL1B; PIK3R5; TLR4; SOCS3
TGF-beta signaling pathway	BMP5; BMP6; DCN; ZFYVE16
Cytosolic DNA-sensing pathway	IL18; IL1B
NOD-like receptor signaling pathway	IL18; IL1B
MAPK signaling pathway	CACNA1D; FGF14; IL1B; NRK; PLA2G4A; PRKCB; PTPN5; RAC2, DUSP5; FOS; PLA2G4A, CACNA1D; CACNA2D1
Herpes simplex infection	CD74; HLA-DRA; IFNGR1; IL1B; TLR2A; FOS; SOCS3
Salmonella	FOS, PKN3
Cell adhesion molecules (CAMs)	SIGLEC1, CD80; VCAM1

*Lactobacillus acidophilus* inhibits the growth of pathogenic bacteria in the gut of poultry and modulates the immune status of poultry birds (Lin et al. 2008). The impact of *Lactobacillus acidophilus* on growth performance and intestinal health of broiler chickens affected with *CP* was evaluated. The use of *Lactobacillus acidophilus* as probiotic increased the weight gain and decreased mortality. It was further concluded that it decreased the intestinal lesion of *CP* challenged birds, ileal populations of *Escherichia coli* and endotoxin quantity in the blood (Li et al. 2018). *Lactobacillus fermentum* and *Lactobacillus acidophilus* were investigated as potential probiotic for treatment of birds infected with *CP*. This *Lactobacillus* spp. inhibited the growth of *CP*, α toxin production and decreased the attachments of *CP* to cells (Guo et al. 2017).

### Miscellaneous microbes used as probiotic for prevention of subclinical necrotic enteritis

The use of *Enterococci* for prevention of SNE is attributed to their ability to produce acids and hydrogen peroxide (Klose et al. 2010). *Enterococcus* bacteria also release bacteriocins called enterocins, which have anti-*CP* activity. Enterocins type A and B produced by *Enterococcus faecium*, inhibit the growth of *CP* (Shin et al. 2008). *Enterococci* have the ability to inhibit toxin production by *CP*. Different *Enterococci* which have shown anti-*CP* activity include *Enterococcus faecium* (Klose et al. 2010), *Enterococcus faecalis* (Fukata et al. 1991) and *Enterococcus durans* (Caly et al. 2015). Administration of probiotic *Enterococcus faecium* NCIMB 1118 significantly alleviated body weight loss, intestinal lesions, histopathological inflammation and intestinal-cell apoptosis in NE challenged birds. Thus, *Enterococcus faecium* NCIMB 1118 reduced the intestinal barrier injury in broilers having NE (Wu et al. 2019). *Butyricicoccus pullicaecorum* strain 25-3T acts as potential probiotic by reducing the abundance of potentially important pathogens in the caeca and ileum (Eeckhaut et al. 2016). Schoster et al. (2013) demonstrated that *Bifidobacteria animalis sub species lactis*, which is a commercial strain, has an antagonistic activity for *CP*. It produces narrow spectrum molecules, which inhibit the growth of *CP*. *Bifidobacteria thermoacidophilum* isolated from the porcine intestine can also be used for prevention of SNE. Lactate and hydrogen peroxide production is the mechanism by which it prevents the growth of *CP* (Klose et al. 2010). *Clostridium butyricum* YH108 as a probiotic showed significant improvement in immune response and intestinal barrier function in chickens affected with necrotic enteritis (Huang et al. 2019).

### Yeast as probiotics for prevention against SNE

Yeast products are important natural growth promoters. *Saccharomyces cerevisiae* was first reported yeast used as a growth promoter for animals. Probiotics that constitute various yeast products efficiently improve the production potential and immune indices of animal and even human beings by optimizing the microbial balance (Awais et al. 2019). Commercial yeast products specifically for animal feeds are used worldwide (Eckles and Williams 1925). The yeast cell wall consists of protein, glucans, and mannan. In poultry, yeast as a probiotic has more effect of improving the performance of birds. *Saccharomyces cerevisiae*, obtained from intestine of poultry, functions as an effective probiotic for prevention of NE (Layton et al. 2013). Priya and Babu (2013) showed that *Saccharomyces cerevisiae* supplementation had a positive effect on the morphology of intestine.

The diet of broiler chickens, challenged with *CP*, was supplemented with *Saccharomyces cerevisiae* as probiotic. The yeast not only had a positive effect on serum biochemical parameters in broilers but also boosted the immune system of chickens (Al-Baadani et al. 2018). *Butyricicoccus pullicaecorum* as probiotic was investigated on growth performance, the composition of intestinal microflora and resistance against *CP*. The strain not only decreased the number of necrotic lesions in chickens infected with necrotic enteritis but also improved the growth performance. It was concluded that *Butyricicoccus pullicaecorum* could be used as a potential valuable probiotic for prevention of necrotic enteritis (Eeckhaut et al. 2016). *Saccharomyces boulardii*, when used as a probiotic, antagonizes the growth of *Candida albicans*. It secretes capric acid, which inhibits colonization, adhesion and biofilm formation of *Candida albicans* (Krasowska et al. 2009; Murzyn et al. 2010). *Saccharomyces boulardii* was evaluated as probiotic to examine its antibacterial potential. It reduced caecal *Salmonella* colonization in broiler chicks. Interestingly, *Saccharomyces boulardii* did not inhibit *Campylobacter colonization* in the same group of broiler chickens (Line et al. 1998). Poultry birds when fed 2.5 g yeast culture/kg feed, modulated villus height to crypt depth ratios in the small intestine, improved digestibility of calcium and phosphate and soared the IgM and secretory IgA concentrations in the duodenum (Gao et al. 2008).

### Molecular mechanisms explaining the positive effects of probiotics against SNE

**Fig. 2:**
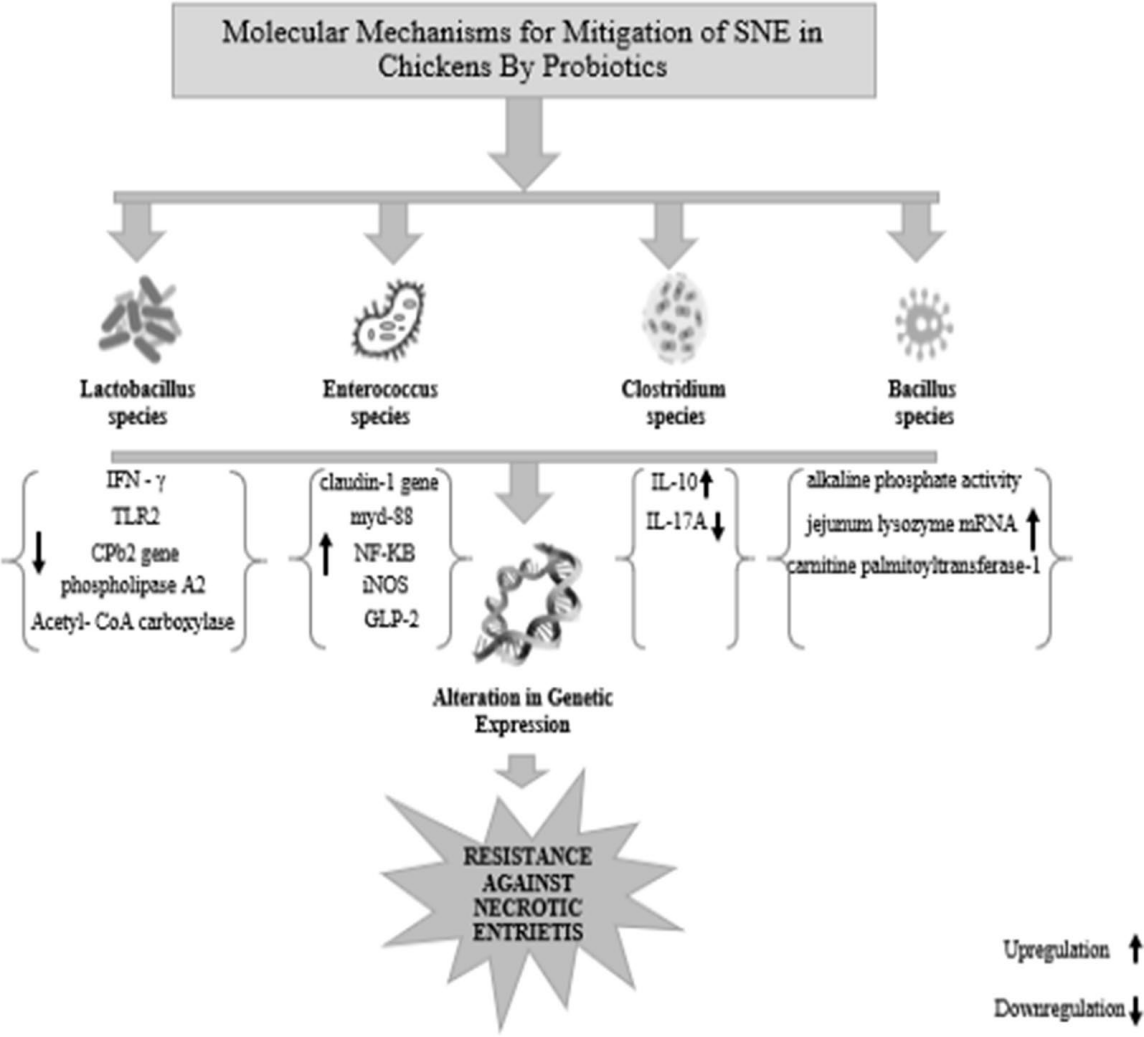
Molecular mechanisms explaining the positive effects of probiotics against SNE

Addition of *Enterococcus faecium* NCIMB 11181 to poultry feed as a probiotic upregulates the expression of the Claudin-1 gene encoding a tight-junction protein in necrotic enteritis affected chickens. Probiotic also leads to an increase in the expression of MyD88, NF-κB, iNOS, PI3K, GLP-2, IL-1β, IL-4, and HSP70 mRNA (Wu et al. 2019). Diets supplemented with *Clostridium butyricum* YH108 have a positive effect on the gut microbiota, immune response and intestinal barrier function in chickens affected with necrotic enteritis. *Clostridium butyricum* not only increased the expression of anti-inflammatory IL-10 in infected chickens but also inhibited the expression of IL-17A gene and reduction of Claudin-1 gene in infected chickens (Fig. 2) (Huang et al. 2019). *Candidatus savagella* induces the formation of antibodies in SNE affected chickens by stimulating T-helper cells (Sokale et al. 2019). *Lactobacillus fermentum* and *Lactobacillus acidophilus* decrease the expression of pro-inflammatory cytokines in birds affected with NE (Wang et al. 2018). *Lactobacillus acidophilus* was responsible for the decline in RNA expression of pro-inflammatory cytokines in birds affected with *CP* (Qing et al. 2017). By decreasing cpb2 mRNA, *Lactobacillus fermentum* prevented the β toxin production by *CP* (Allaart et al. 2011). By increasing IL-10 mRNA levels and decreasing the mRNA expression of IFN- γ and TLR2, *Lactobacillus fermentum* reduced the inflammatory damage caused by *CP* induced NE (Cao et al. 2012). *Lactobacillus johnsonii BS15* increased the expressions of fatty acid-binding protein 2, acyl-CoA synthetase bubblegum family member 1, perilipin 1 and perilipin 2 and suppressed phospholipase A2 group IVA in arachidonic acid metabolism (Qing et al. 2018). *Lactobacillus johnsonii BS15* prevented subclinical necrotic enteritis by reducing expression of genes expression of acetyl-CoA carboxylase, fatty acid synthase and sterol regulatory element-binding protein-1c. It also enhanced gene expression of peroxisome proliferator-activated receptor α and carnitine palmitoyltransferase-1 while helped in mitigating subclinical necrotic enteritis (Qing et al. 2017). *Lactobacillus johnsonii BS15* significantly increased in the CD3^+^CD4^+^ T-lymphocyte percentage in peripheral blood of birds affected with SNE. It also increased the serum levels of immunoglobulins and cytokines that were affected by SNE (Cao et al. 2012). *Lactobacillus johnsonii BS15* improved intestinal immunity in broilers against subclinical necrotic enteritis by positively altering mRNA expression levels of apoptosis-related proteins (Wang et al. 2017). *Bacillus coagulans* when fed as probiotic to SNE affected birds led to increase in the IgA levels, alkaline phosphatase (IAP) activity in the jejunum and the expression of jejunum lysozyme mRNA (Wu et al. 2018).

*Bacillus licheniformis* helped to normalize the ileum microbiota of chickens infected with SNE by positively correlating a tumour suppressor gene, p53 (Xu et al. 2018). *Bacillus licheniformis* significantly upregulated catabolism-related genes, i.e. peroxisome proliferator-activated receptor-α and carnitine palmitoyltransferase-1 in livers in SNE affected birds. It also altered the expression of lipid-anabolism genes (Zhou et al. 2016).

## Conclusion

SNE caused by *CP* causes gigantic economic loses in terms of growth and production performance in poultry. Due to the ban of antibiotics in poultry feed, probiotics are best available alternatives to limit the intestinal inflammation caused by *CP*. Different microorganisms like *Bacillus*, *Lactic acid bacteria*, *Bifidobacteria*, *Enterococcus*, and yeast have been investigated as potential probiotics. The beneficial effects of many of the developed probiotics have been well demonstrated in the prevention of SNE in chickens. The general consensus is that the outcome and results of these probiotics may vary under farm conditions. Furthermore, the mode of action of these probiotics needs to be better understood. To achieve the ultimate goal of reducing or preventing antibiotic use in the poultry feed as a growth promoter. Therefore, it is an urgent need to develop new probiotics which are practically more feasible under farm conditions.

## Data Availability

Not applicable.
